# Socio-demographic and toxicological findings from autoptic cases in a Northern Italy community (2017–2022)

**DOI:** 10.1007/s00414-025-03433-1

**Published:** 2025-02-03

**Authors:** Federica Palazzoli, Tommaso Filippini, Antonino Lavenia, Simone Balduini, Alessia Attanasi, Patrizia Verri, Daniele Vandelli, Valentina Castagnetti, Anna Laura Santunione, Marco Vinceti, Rossana Cecchi

**Affiliations:** 1 Institute of Legal Medicine, Department of Biomedical, Metabolic and Neural Sciences, University of Modena and Reggio Emilia, Modena, Italy; 2 CREAGEN - Environmental, Genetic and Nutritional Epidemiology Research Center, Section of Public Health, Department of Biomedical, Metabolic and Neural Sciences, University of Modena and Reggio Emilia, Modena, Italy; 3 School of Public Health, University of California Berkeley, Berkeley, CA USA; 4 Department of Epidemiology, Boston University School of Public Health, Boston, MA USA

**Keywords:** Forensic toxicology, Post-mortem, Benzodiazepines, Drug abuse, Licit drug, Illicit drug

## Abstract

**Introduction:**

The overall trend in the use of licit and illicit substances is increasing. However, a few data on socio-demographic and toxicological findings in post-mortem cases are available.

**Methods:**

A retrospective study was conducted on autoptic cases evaluated in the Institute of Legal Medicine of the cities of Modena and Reggio Emilia in the period 2017–2022. Positivity to toxicological compounds and their relation with sociodemographic and forensic features were evaluated.

**Results:**

A toxicological analysis was conducted in 504 cases out of 794 autopsies, finding 330 positive cases. An association was observed between positivity and increasing age, as well as manner of death. The most frequently observed classes of substances were benzodiazepine (41.2%), followed by alcohol (35.5%) and abuse drugs (24.8%). For every class of substances in at least half of the cases two or more classes were detected. As regards prescribed drugs, approximately 40% of cases assumed 2 or more drugs, while polypharmacy occurred in 6.1%.

**Conclusions:**

Older age and specific causes of deaths appear to be associated with toxicological findings. In addition, co-assumption of licit and illicit substances emerged as a widespread phenomenon in our study population. Under a public health perspective, these data provide findings of relevance for preventive and therapeutic measures.

**Supplementary Information:**

The online version contains supplementary material available at 10.1007/s00414-025-03433-1.

## Highlights

- The overall trend in the use of licit and illicit substances is increasing.
- Benzodiazepines, alcohol and abuse drugs are the most frequently detected substances.
- Age and specific manner of death are associated with positive toxicological findings.
- This study provides a scientific basis for preventive policies and care strategies.

**Supplementary Information:**

The online version contains supplementary material available at 10.1007/s00414-025-03433-1.

## Introduction

Forensic toxicology is an ancillary branch of forensic medicine that nowadays plays an increasingly central role in forensic medical practice. Results from toxicological investigations could provide important information about the characteristics of examined populations, since social, cultural and economic factors could may be involved in in the prevalence of licit and illicit drug use [1, 2]. In Italy, the abuse of alcohol, drugs and other substances is still a rather widespread phenomenon [3–6]. Medications are even more widespread as more than 6 in 10 citizens received at least one drug prescription in 2022 [7]. Knowledge and analysis of forensic toxicology cases in a specific area allow local government to improve public health with prevention and control projects [8, 9].

At present, a number of studies conducted in specific areas of Italy have reported retrospective analyses of specific topics including drug-facilitated crimes [10], complex suicides [11], acute intoxication fatalities [12] or toxicological analyses performed over a two-year period [13]. Based on an analysis of cases from Milan over a restricted two-year period (2018–2019), in particular, Di Candia et al. identified a typical subject dying of acute intoxication: a Caucasian Italian male (age range 41–50) who died from acute cocaine intoxication. Pelletti et al. estimated the incidence of licit psychoactive drugs in cases of victims of drug-facilitated crimes and acute intoxication in Bologna between 2013 and2017, reporting an increased assumption of licit drugs in complex suicides. In a study assessing complex and complicated suicides in Genoa between 2006 and 2017, on the other hand, Barranco et al. [11] found only one death from a combination of benzodiazepines, alcohol and wrist cutting. In an eight-year study (2009–2016) from Parma, Anzillotti et al. [12] analyzed post-mortem toxicological findings in order to assess changes over time in drug death rates, causes and circumstances. The authors highlighted heroin injection as a major cause of death in acute intoxication cases, while alcohol is the most widely detected substance involved in abuse. Since most of the previous studies are characterized by a limited number of cases, or they are restricted to specific populations (e.g., victims of crimes, acute intoxication, complex suicides) and based on thorough toxicological evaluation, this study is aimed at a better understanding of post-mortem social-demographic traits and toxicological characteristics of the population in the provinces of Modena and Reggio Emilia.

## Methods

### Study design

All autopsies (857 cases) performed between 2017 and 2022 and referred to the morgue of the Institute of Legal/Forensic Medicine of the University of Modena and Reggio Emilia were included in the study. The Institute is the reference point for the cities of Modena and Reggio Emilia and their provinces, covering over 1,23 million inhabitants in the period considered [14, 15]. For all autopsy cases, data were collected from internal registers about sex, age, ethnicity, nationality, place of death (i.e. house, public space, place of work, and place of confinement), death timing, corpse conservation state (i.e. early changes, decomposition, carbonization, skeletal stage and mummification), and manner, cause and mechanism of death. Dates of death were further categorized into seasons according to meteorological classification [16]. Manner of death was divided into four groups (natural, accidental, suicidal and homicidal deaths), also classifying each case according to cause (e.g., traffic accident, intoxication and gunshot wounds) and mechanism of death (e.g., cardiac death, cerebral hemorrhage, traumatic/hemorrhagic shock).

### Forensics

#### Autoptic procedure

Autopsies followed a standardized protocol, including an examination of all organs through a sequential approach. When indicated, forensic toxicological evaluation was carried out. Autopsies were performed from 24 h to 10 days after declaration of death, generally within 48–72 h. Corpses were kept at −20 or 0 °C depending on time between declaration of death and autopsy.

#### Toxicological analysis

Toxicological analyses were requested in 504 cases (63%) to establish the presence of any xenobiotics evaluate their role as determinants or contributors to death. In general, toxicological investigations included ethanol (and volatiles substance) determination along with a general screening for licit drugs and illicit substances (a total of 370 substances). For toxicological analyses, all biological samples collected were stored in polystyrene tubes with sodium fluoride (1% w/v) at −20 °C. As a routine practice, central blood (taken from the heart), peripherical blood (taken from the femoral vein), urine, bile, kidney, liver, brain and gastric contents were collected. When necessary, also samples from hair, vitreous humor and lungs were used for toxicological assessment. A systematic toxicological analysis (STA) was carried out following a standard protocol: blood and urine were screened using EMIT immunoassay for amphetamines, ecstasy, barbiturates, benzodiazepines, buprenorphine, methadone, cocaine, cannabinoids, opiates and valproic acid. Moreover, a comprehensive screening for pharmaceuticals and drugs of abuse, including New Psychoactive Substances (NPS) and fentanyl analogues, was performed on fluid samples and tissue specimens using liquid chromatography-tandem mass spectrometry (LC–MS/MS) and full scan gas-chromatography-mass spectrometry (GC–MS). A quantitative analysis was performed by specific calibration curve, using appropriate working standard solutions and internal standard solutions. To detect ethanol and other volatile substances, GC-HS-FID (head-space gas chromatography with flame ionization detection) in peripheral blood was used and, when unavailable, central blood and/or brain samples were also used. Carboxyhemoglobin (HbCO) levels were evaluated in central blood immediately after sampling through the Conway cell microdiffusion method followed by ultraviolet–visible spectrophotometer (UV–Vis) analysis. Potassium analysis was carried out using a Varian atomic absorption spectrometer (FS220 series, California, USA). Positive alcohol findings are in keeping with the detection of ethanol in concentrations above the limit of quantification (LOQ) of 0.1 g/L (or g/kg), while positive drug findings comprise detection of all other exogenous drugs in concentrations above the limit of quantification LOQ of the specific xenobiotic.

**Fig. 1:**
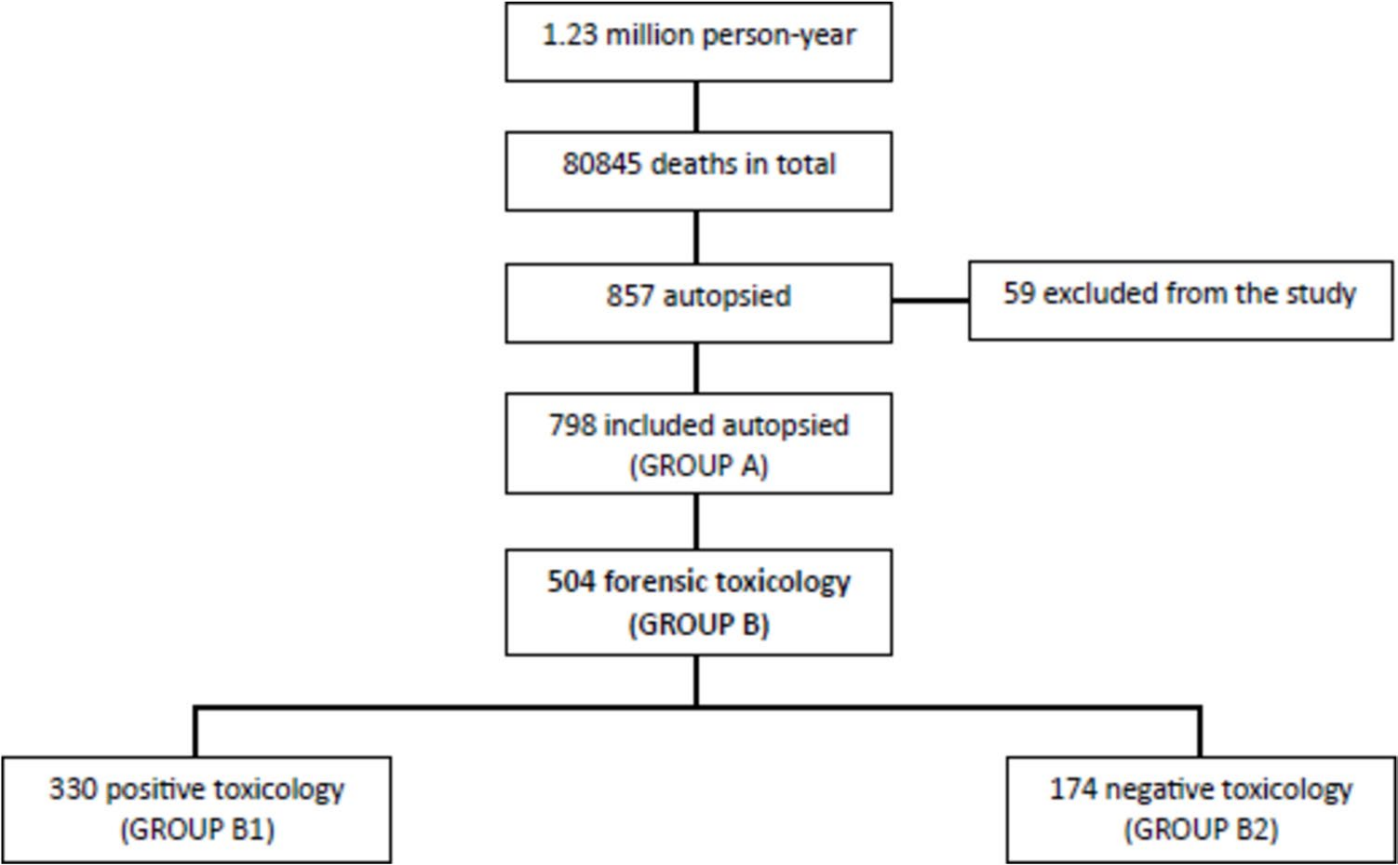
Flow chart with autopsy cases from deceased persons between 2017 and 2022 in Modena and Reggio Emilia, Northern Italy

Based on the presence or absence of toxic and pharmaceutical substances, cases were divided into two groups: positive (group B1) and negative (group B2) toxicology (Fig. 1). Toxicology assessment was defined as ‘positive’ in the presence of at least one of the following categories of substances: ethanol, carbon monoxide, benzodiazepines, antipsychotics, antidepressants, anticonvulsants, opioid agonists, drugs of abuse, antihypertensives and others (Supplementary Table S1). Supplementary Table S1 reports the limit of detection (LOD) and lowest limit of quantification (LLOQ) of the analytical methodology. Morphine administered by healthcare personnel or taken as homecare therapy was included among opioid agonists. Paracetamol and ibuprofen were not considered in this study in that they are widespread and generally without toxicological interest. The gold standard to determine the state of intoxication of an individual is the detection and quantification of substances in femoral blood and the brain [17]. Toxicological data were evaluated by taking account of all other medical legal criteria (clinical-anamnestic, circumstantial, anatomo-histopathological). The presence of five or more concurrent drugs was defined as “polypharmacy” [18].

### Statistical analysis

Summary statistics was used for categorical variables through absolute and relative frequencies, while mean and standard deviation (SD) were used for continuous variables. Odds ratio (OR) was computed along with its 95% confidence interval (CI) of overall and specific toxicological positivity through unconditional logistic regression analysis. All analyses were performed through crude and adjusted models including sex, age (divided into ≤ 30, > 30 and ≤ 60, and > 60 years), nationality (Italians and Non-Italians), place of death, season, and manner of death. Excel (Office Package, Microsoft) and StataNowMP v18.5 (StataCorp LCC, College Station, TX, 2023) were used for all data analysis.

## Results

Overall, 857 autopsies were performed in the study period. Fifty-nine cases were excluded from the study due to a lack of relevant information in autopsy reports. The demographic and circumstantial characteristics of the 798 cases included are shown in Table 1. Most cases were males (70.7%) and of Italian nationality (77.4%), with a mean age of 55.6 (SD: 17.2) years, with half of the subjects aged between 30–60 years (52%) followed by those above 60 years (41%). As for manner of death, natural death (66.9%) was the most common, followed by accidental deaths (21.7%), suicide (6.9%) and homicide (4.5%). As far as mechanism of death is concerned, cardiac death was the most common (53.5%), followed by traumatic/hemorrhagic shock (17.9%). The remaining causes of death were asphyxia (8.0%), MOF (Multi Organ Failure) (7.9%), cerebral/pulmonary edema (4.6%), cerebral hemorrhage (4.5%), pulmonary embolism (2.8%) and others (0.7%). Among unnatural deaths, traffic accident (27.3%) prevailed, followed by acute intoxication (25.8%) and asphyxia deaths (17.1%). The most common place of death was a private house (67.3%), followed by public space (18.7%), health facility (8.2%), workplace (33.0%) and place of confinement (1.8%). In terms of corpse conservation state, the majority of the individuals were characterized by early changes (79.5%), followed by decomposition (19.3%), carbonization (1.0%), skeletal stage and mummification (one case each, only). Toxicological analyses were requested in 504 cases (63.0%): 330 (65.5%) had a positive toxicological profile, while 174 (34.5%) were negative. Toxic substances were considered the leading cause of death in 68 cases (13.5%).

**Table 1:** Characteristics of study subjects. Data are numbers and percentages, unless otherwise indicated

	*Overall* *(n* = *798)*	*Without toxic investigation (n* = *294)*	*With toxic investigation* *(n* = *504)*	*Positive Tox* *(n* = *330)*	*Negative Tox* *(n* = *174)*
*Sex*					
Male	564 (70.7)	204 (69.4)	360 (71.4)	234 (70.9)	126 (72.4)
Female	234 (29.3)	90 (30.6)	144 (28.6)	96 (29.1)	48 (27.6)
*Age*					
Mean ± SD	55.6 ± 17.2	62.2 ± 15.7	51.7 ± 16.9	52.1 ± 16.0	51.0 ± 18.5
≤ 30 years	56 (7.0)	8 (2.7)	48 (9.5)	26 (7.9)	22 (12.6)
> 30 ≤ 60 years	415 (52.0)	112 (38.1)	303 (60.1)	206 (62.4)	97 (55.8)
> 60 years	327 (41.0)	174 (59.2)	153 (30.4)	98 (29.7)	55 (31.6)
*Nationality*					
Italians	618 (77.4)	246 (83.7)	372 (73.8)	240 (72.7)	132 (75.9)
Non-Italians	180 (22.6)	48 (16.3)	132 (26.2)	90 (27.3)	42 (24.1)
*Place of death*					
House	537 (67.3)	216 (73.5)	321 (63.7)	209 (63.3)	112 (64.4)
Public space	149 (18.7)	36 (12.2)	113 (22.4)	73 (22.1)	40 (23.0)
Health facility	65 (8.2)	35 (11.0)	30 (6.0)	23 (7.0)	7 (4.0)
Workplace	33 (4.1)	6 (2.0)	27 (5.4)	13 (3.9)	14 (8.1)
Place of confinement	14 (1.8)	1 (0.3)	13 (2.6)	12 (3.6)	1 (0.6)
*Season*					
Spring	204 (25.6)	76 (22.8)	128 (25.4)	83 (25.2)	45 (25.9)
Summer	198 (24.8)	67 (22.8)	131 (26.0)	95 (28.8)	36 (20.7)
Autumn	188 (23.6)	75 (25.5)	113 (22.4)	65 (19.7)	48 (27.6)
Winter	208 (26.1)	76 (22.8)	132 (26.2)	87 (26.4)	45 (25.9)
*Cadaveric state*					
Early changes	634 (79.5)	243 (82.7)	391 (77.6)	242 (73.3)	149 (85.6)
Decomposition	154 (19.3)	50 (17.0)	104 (20.6)	81 (24.6)	23 (13.2)
Carbonized	8 (1.0)	1 (0.3)	7 (1.4)	5 (1.5)	2 (1.2)
Skeletal stage	1 (0.1)	0 (0.0)	1 (0.2)	1 (0.3)	0 (0.0)
Mummification	1 (0.1)	0 (0.0)	1 (0.2)	1 (0.3)	0 (0.0)
*Mechanism of death*					
Cardiac death	427 (53.5)	178 (60.5)	249 (49.4)	154 (46.7)	95 (54.6)
Traumatic/Hemorrhagic shock	143 (17.9)	34 (11.6)	109 (21.6)	60 (18.2)	49 (28.2)
Asphyxia	64 (8.0)	10 (3.4)	54 (10.7)	44 (13.3)	10 (5.8)
Multi Organ Failure	63 (7.9)	36 (12.2)	27 (5.4)	17 (5.2)	10 (5.8)
Cerebral hemorrhage	36 (4.5)	20 (6.8)	16 (3.2)	10 (3.0)	6 (3.5)
Pulmonary embolism	22 (2.8)	13 (4.4)	9 (1.8)	6 (1.8)	3 (1.7)
Cerebral/pulmonary edema	37 (4.6)	0 (0.0)	37 (7.3)	37 (11.2)	0 (0.0)
Others	6 (0.8)	3 (1.0)	3 (0.6)	2 (0.6)	1 (0.6)
*Manner of death*					
Natural Cause	534 (66.9)	246 (83.7)	288 (57.1)	170 (51.5)	118 (67.8)
Accidental	173 (21.7)	28 (9.5)	145 (28.8)	112 (33.9)	33 (19.0)
Suicide	55 (6.9)	9 (3.1)	46 (9.1)	35 (10.6)	11 (6.3)
Homicide	36 (4.5)	11 (3.7)	25 (5.0)	13 (3.9)	12 (6.9)

### Negative toxicological findings

Negative results were detected in 174 of 504 cases for which toxicological investigations were carried out between 2017–2022. The population was composed of 48 females and 126 males A majority of the individuals were Italians (75.9%). Death occurred in autumn in 48 cases (27.6%), followed by spring and winter (25.9% each), and summer (20.7%). Mean age was 51.0 ± 18.5. The age group between 30 and 60 years (55.8%) was the most widely represented. The most common cause of death in these negative cases was natural death (67.8%), followed by accidental death (19.0%), homicide (6.9%) and suicide (6.3%). The most common cadaveric state was early changes (85.6%). No skeletal and mummification stages have been recorded. The most common place of death was a private house (64.4%), followed by public space (23.0%), workplace (8.1%), health facility (4.0%) and place of confinement (only onecase).

### Positive toxicological findings

Positive results were detected in 330 of the 504 toxicological investigations. These cases included 234 males (70.9%) and 96 females (29.1%), mostly Italians (72.7%). A larger share of death in private house settings (63.3%) emerged, followed by public space (22.1%), health facility (7.0%), workplace (3.9%) and, finally, place of confinement (3.6%). Occurrence of death was slightly higher in the summer (28.8%) than in winter (26.4%) and spring (25.2%), and far more higher than in the autumn (19.7%). The manner of death in toxicological positive cases was mainly natural death (50.0%), followed by accidental death (35.2%), suicide (10.9%) and homicide (3.9%). As far as cadaveric state, the toxicological analysis were reported in cases of early changes (73.3%), followed by decomposition (24.6%).

Table 2 summarizes the association between risk of positive toxicological findings and relevant demographic and circumstantial characteristics, in both crude and adjusted analyses. No association emerged for sex, while a positive association with increased ORs was reported for the 30–60 age group and, in part, for those > 60 years compared to those aged < 30 years. As regards place of death, we found a positive association with residence in health facilities and places of confinement, and a strong inverse association with workplace. A strong positive association was found for accidental death and suicide in terms of manner of death. In the multivariable model, the correlations in the unadjusted analysis were generally confirmed and strengthened.

**Table 2:** Odds ratio (OR) with 95% confidence interval of risk of positive toxicological findings according to demographic and circumstantial characteristics in tested subjects (*N* = 504)

	*Crude*			*Adjusted*		
	*OR*	*95% CI*	*P value*	*OR*	*95% CI*	*P value*
*Sex*						
Male	1.00	-		1.00	-	
Female	1.08	(0.72–1.62)	0.722	1.16	(0.74–1.83)	0.512
*Age*						
≤ 30 years	1.00	-		1.00	-	
> 30 ≤ 60 years	1.80	(0.97–3.33)	0.063	2.09	(1.07–4.10)	0.031
> 60 years	1.51	(0.78–2.91)	0.221	1.83	(0.90–3.74)	0.096
*Nationality*						
Italians	1.00	-		1.00	-	
Non-Italians	1.18	(0.77–1.80)	0.447	1.13	(0.71–1.79)	0.606
*Place of death*						
House	1.00	-		1.00	-	
Public space	0.98	(0.62–1.53)	0.923	0.74	(0.45–1.23)	0.248
Health facilities	1.76	(0.73–4.23)	0.206	1.46	(0.59–3.65)	0.415
Workplace	0.50	(0.23–1.10)	0.083	0.31	(0.13–0.74)	0.008
Place of confinement	6.43	(0.83–50.10)	0.076	4.19	(0.51–34.17)	0.181
*Season*						
Spring	1.00	-		1.00	-	
Summer	1.43	(0.84–2.43)	0.184	1.50	(0.86–2.60)	0.149
Autumn	0.73	(0.44–1.24)	0.244	0.78	(0.45–1.35)	0.380
Winter	1.05	(0.63–1.75)	0.857	1.11	(0.65–1.89)	0.712
*Manner of death*						
Natural Cause	1.00	-		1.00	-	
Accidental	2.36	(1.50–3.71)	< 0.001	3.00	(1.79–5.04)	< 0.001
Suicide	2.21	(1.08–4.52)	0.030	2.18	(1.04–4.55)	0.039
Homicide	0.75	(0.33–1.71)	0.495	0.82	(0.35–1.96)	0.659

Adjusted by sex, age category, nationality, place of death, season, and manner of death

**Fig. 2:**
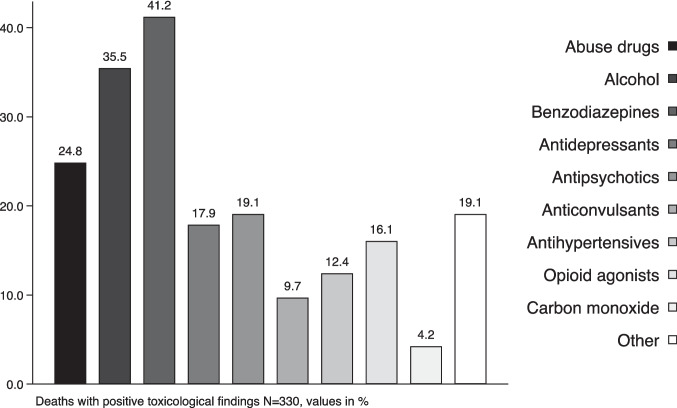
Distribution of positive toxicological findings. Different groups of compounds are depicted using decreasing grey-scale patterns and in the order of the legend

Overall, 139 different analytes were detected in toxicologically positive cases. These substances were subdivided into the followed classes: ethanol, carbon monoxide, benzodiazepines, antipsychotics, antidepressants, anticonvulsants, opioid agonists, drugs of abuse, antihypertensive and others (Fig. 2). The most frequently observed classes of substances were benzodiazepine (41.2%), followed by alcohol (35.5%) and drugs of abuse (24.8%). Full details about the detected substances are reported in Supplementary Table S2.

**Fig. 3:**
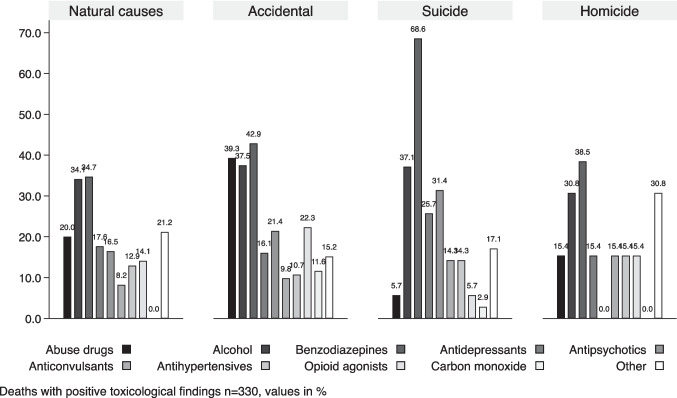
Distribution of positive toxicological findings divided by manner of death. Different groups of compounds are depicted using decreasing grey-scale patterns and in the order of the legend

Figure 3 shows the distribution of the identified classes across groups in relation to manner of death: the most widely detected class of substances was benzodiazepines, with a prevalence of 68.6% in suicide cases, followed by accidental deaths (42.9%), homicide (38.5%) and natural deaths (34.7%). Drugs of abuse were most frequently found in accidental deaths (39.3%), while the lowest number was found in suicides (5.7%).

Relative to alcohol class, no substantial differences between manner of deaths groups have been highlighted (Fig. 3).

**Fig. 4:**
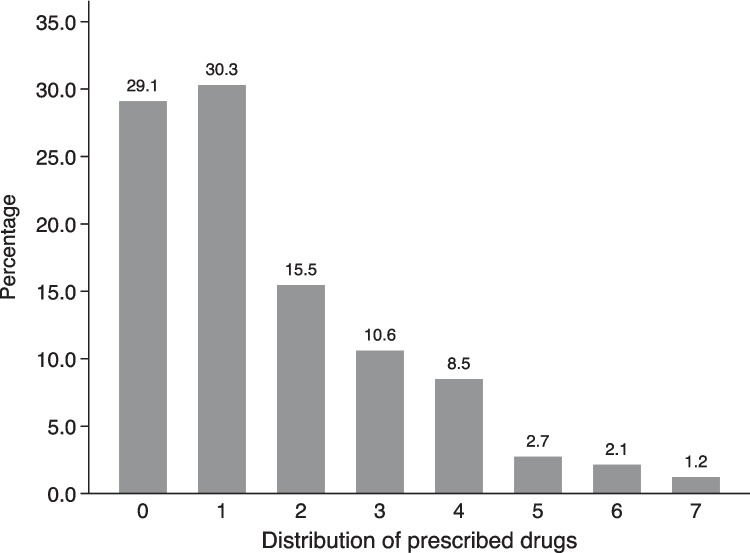
Distribution of number of prescribed (licit) drugs in positive cases (N=330). The numbers in the x-axis represent the simultaneous occurrence of prescribed drugs

The occurrence of two or more classes of substances was also investigated. Supplementary Figure S1 shows the co-occurrence rates of the investigated toxicological substances divided by main types and number of classes. For all classes of substances, two or more classes were detected in at least half of the samples. As regards licit (prescribed) drugs, the percentage of the number of different substances detected at the same time in positive cases (poly-assumption) is reported in Fig. 4. In approximately 40% of cases, the subject assumed 2 or more drugs with a median value of 1 drug (IQR: 0–3). Polypharmacy (i.e. ≥ 5 drugs) occurred in 6.1% of cases (*N* = 20). Supplementary Figure S2 reports the distribution of licit drugs stratified by sex and age categories. Slightly lower median values were found in males (*N* = 1, IQR: 0–2) compared to females (*N* = 1.5, IQR: 1–3), but with 6.8% (*N* = 16) and 4.2% (*N* = 4) of polypharmacy, respectively. Analysis divided by age demonstrated that younger subjects (< 30 years) generally assumed less prescribed drugs compared to older individuals (Supplementary Figure S3).

**Fig. 5:**
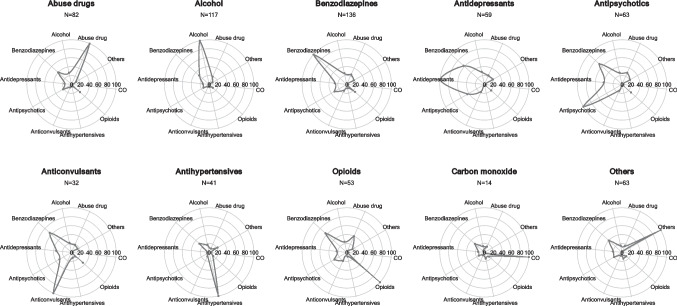
Spider plots of xenobiotic positivity divided by main classes of substances. For each xenobiotic, the percentage of co-occurrence of other classes is reported

Figure 5 presents spider plots with rates of contemporary detection for the investigated classes. Except for antihypertensives and carbon monoxide, multi-detection of classes of substances can be noted. In particular, benzodiazepines were detected in 30% of all cases, with higher rates over 60% in association with antidepressants, antipsychotics, anticonvulsants, and opioids. In addition, benzodiazepines have been detected alongside alcohol in more than 20% of all cases. Similarly, both antipsychotics and antidepressants (though with lower rates of co-detection compared to benzodiazepines) were found in over 30% of subjects who are also positive to benzodiazepines, antidepressants, anticonvulsants and opioids.

Toxic substances were neither considered as the first cause nor a fundamental cause of death in 256 cases, while the cause of death was ascertained as fatal acute intoxication in 68 cases thanks to toxicological findings (13.5% of the total number of cases with toxic investigation and 20.6% of the positive cases).

Supplementary Table S2 outlines drugs and illicit substances detected through toxicological analysis. In terms of individual analytes, ethanol was found in 117 cases (23.2% of all cases and 35.5% of all toxicologically positive cases), benzoylecgonine in 60 cases (11.9%—18.2%) and lorazepam in 54 cases (10.7%—16.4%).

Among illicit drugs, the most highly consumed substance was cocaine (*n* = 54, 10.7%—16.4%; benzoylecgonine: *n* = 60, 11.9%—18.2%), followed by heroin (6-MAM: *n* = 31, 6.2%—9.4%; morphine: *n* = 38, 7.5%—11.5%) and cannabis (11-Nor-9-carboxy-delta-9-tetrahydrocannabinol: *n* = 22, 4.4%—6.7%, delta-9-tetrahydrocannabinol *n* = 19, 3.8%—5.8%, and 11-Hydroxy-delta-9-tetrahydrocannabinol *n* = 16, 3.2%—4.8%). Additionally, cocaethylene was also detected *n* = 34, 6.7%—10.3%). Among benzodiazepines, lorazepam (*n* = 54, 10.7%—16.3%) and diazepam (*n* = 34, 6.7%—10.3%) were the most widespread. Among antipsychotics, the most widely used was quetiapine (*n* = 24, 4.8%—7.3%), while trazodone and citalopram were the most frequently detected antidepressants, retrieved in 15 (3.0%—4.5%) and 13 cases (2.6%—3.9%), respectively. Among opioid agonists, the most widely used was methadone (*n* = 24, 4.8%—7.3%). Among anticonvulsants, pregabalin (*n* = 15, 3%—4.5%) was most widespread, as was amlodipine among antihypertensives (*n* = 16, 3.2%—4.8%).

Supplementary Tables S3-S11 present crude and adjusted OR of positivity to classes of toxics, also considering subjects’ demographic and circumstantial characteristics. For drugs of abuse, an inverse association was found for female sex, age > 60 years, and non-Italian nationality. Similarly, all places of death showed a negative association, compared to private house. Conversely, a direct association was present for accidental manner of death.

Detection of alcohol occurrence was inversely associated with female sex and increasing age, and directly with non-Italians. Public space showed a stronger positive association compared to private house and other places of death. Association with manner of death was generally positive, although weaker compared to other characteristics.

Benzodiazepine detection was strongly associated with female sex and age older than 30 years, while it was the opposite with non-Italian nationality. As for places of death, work and public spaces showed inverse association, while a positive one could be noted for health facilities and place of confinement, compared to private house. The autumn season tended to be positively associated with benzodiazepine presence as well as both accidental death and suicide. Antidepressants showed a pattern similar to benzodiazepines, except for a weak negative association with health facility as place of death. They showed no association with season, and a weak direct association with manner of death.

For antipsychotics, a negative association was found for non-Italians and public spaces. Conversely, a strong positive association was noted for place of confinement and both accidental death and suicide. Anticonvulsants showed a weak positive association with female sex and with age > 30 years. Similarly, a positive association could be noted for health facility and place of confinement. All other seasons compared to spring showed a negative association, stronger for winter and summer. A positive association was also found with accidental death, suicide and homicide. Antihypertensives were more commonly detected in non-Italians, individuals aged > 60 years and for deaths occurred in work and public spaces. For all other characteristics, no clear associations emerged. Opioid agonists were more commonly detected in individuals aged more than 30 years, as compared to younger subjects. Places of confinement showed the strongest direct association, as did accidental death. Carbon monoxide was more commonly detected in females, over 60 years and in non-Italians. Carbon monoxide was strongly associated with accidental death. A direct weak association was also observed with the winter season.

## Discussion

This study highlights the high frequency of toxicological findings in an autoptic series of cases within a community from Northern Italy over a six-year period of observation. In approximately two thirds of the cases, toxicological analyses were conducted, in keeping with a study carried in Milan [13]. Toxicological positivity was more common with advancing age and particularly after ≥50 years, as observed elsewhere [7, 19].

Concerning the key factors influencing toxicological positivity, and despite the lack of specific sex differences in overall toxicological results, we found some sex-related differences. In particular, males were found to be at higher risk of drug abuse and polypharmacy, while females were identified as being at risk of use of benzodiazepines, antidepressants and, partly, anticonvulsants. Since this last category also encompasses medications used for anxiety, depression and pain therapy, our finding is not entirely unexpected in light of the higher prevalence of depressive and anxiety disorders in Italian females [7, 20]. Similarly, subjects aged 30–60 years showed a stronger positive association with benzodiazepines, antidepressants and anticonvulsants, in keeping with prevalence data [7, 20]. The higher positivity for opioids in older cases could be related to a higher use of maintenance treatment for drug abuse in people aged > 30 years [21], as well as pain therapy with drugs such as tramadol, oxycodone and morphine [22, 23]. Conversely, we established an opposite trend for drug abuse and alcohol, with a higher risk in younger people [6].

Italian nationality was a risk factor for positivity to drug abuse, benzodiazepines, antidepressants and antipsychotics, while the opposite was true for alcohol and antihypertensives. Such difference in alcohol use may be related to the lower socioeconomic status of non-Italians [24], particularly those from North Africa (Morocco and Tunisia) and the Balkans (Romania and Albania). According to the Italian National Institute of Statistics and the World Health Organization, alcohol consumption is higher in Romanians and Albanians than Italians, but lower in North Africans [25, 26]. The discrepancy observed for North Africans between our study and reports from those two public research organizations can be attributed to the differing study populations. Specifically, our research focuses on deceased individuals, among whom a potential risk factor is low socio-economic status, which is itself associated with alcohol abuse. On the other hand, the two reports refer to the general living population.

As for places of death, the association we found between toxicological positivity and place of confinement, and to a lesser extent residence in health facility, was specifically confirmed for benzodiazepines, antidepressants, antipsychotics, anticonvulsants and opioids. This may be linked to a higher risk of use of medications in hospitalized subjects and convicts, including maintenance treatment as already pointed out [27–29]. Conversely, we found a generally lower risk with workplace, highlighting that this type of place of deaths is generally not related to drug use. Rather, they are mostly natural or accidental deaths more frequently through mechanical injuries like falls or crushes.

As regards manner of death, suicide was linked to a higher risk of use of toxic substances and medications, particularly benzodiazepines, antipsychotics and anticonvulsants. These all have psychoactive effects and thus affect subjects’ behavior and consciousness [30]. Similarly, we found such a direct association also for accidental death, but with the addition of abuse drugs and opioids. With reference to benzodiazepines, this result is in agreement with other studies, which have shown an association between drug misuse and suicidal ideation and attempt [31, 32], as well as with risk of traffic accidents [33], falls [34] and work-related injuries [35].

Season seems to play no specific role in the study population, except for a higher risk for winter found for carbon monoxide. As regards manner of death, all but one (suicide) of our cases were accidental, consistent with the fact that carbon monoxide intoxication is generally related to malfunctioning of heating systems in cold seasons [36].

The most frequently detected classes of substances were benzodiazepines, followed by alcohol and abuse drugs. These data are in keeping with previously reported results [12, 13]. The most widely detected analyte is alcohol, frequently in association with other xenobiotics, followed by cocaine. From the annual report to the Italian Parliament on drug addiction, it recently emerged that cocaine consumption is widespread in the country, with an increased trend over the last decade in seizures, usage among young people, socio-economic impact on care services and cocaine-related offences [6].

Benzodiazepines, antipsychotics and antidepressants are the most strongly represented classes when considering prescribed drugs only, with respective co-detection rates above 60%, 30% and 30% in case of positivity to another licit drug. Benzodiazepine use is heavy in Italy, accounting for 17% of total expenditure in 2022 and 22% of Defined Daily Dose (DDD) [7]. In our population, in particular, the top three benzodiazepines are lorazepam, diazepam and alprazolam. This is in line with the most recent report on drug prescription, which indicates that lorazepam and alprazolam are the most common substances. A significant co-detection rate (around 60%) was found with opioids of licit and illicit use. Indeed, Tubbs et al. found that persons who consume opioids were at least 4 times more likely to use benzodiazepines than persons who did not [37]. This association could be explained by the ability of benzodiazepines to increase the positive subjective effects of opioids [38]. Among antipsychotics, quetiapine is the most widely detected medication. More specifically, it is the third antipsychotic for expenditure and DDD in Italy [7].

Figure 5 and Supplementary Figure S1 show the consistency of phenomena such as co-assumption of substances and polypharmacy, which are widespread in Italy. Other studies have already pointed out how commonly more than five medications are co-prescribed, especially in the elderly population [39]. 

The present study is larger and therefore more statistically precise compared with previous studies. It encompasses a larger number of death types, including complex suicides or death from drug-facilitated crimes [10–13, 40]. Finally, the study included a more comprehensive toxicological assessment. As a matter of fact, we used a large number of matrices, i.e. blood and urine samples, but also brain, liver and kidney specimens. In addition, our toxicological investigation was not based on immunoassay analysis, but on multitarget screening also characterized by a very low limit of detection, an approach that allowed for the detection of a wide number of xenobiotics.

Some limitations of this study should be noted. First of all, the deaths examined in the study are not necessarily representative of all deaths occurring in the underlying population. In fact, the choice whether to perform an autopsy, under Italian law, is based on requests from Judicial Authority (judicial autopsies) when a crime is suspected, or from the Health Authority (administrative autopsies) in order to establish the cause of death. Indeed, some differences between cases without and with toxicological investigation are worthy to mention, including a slightly higher mean age (62.2 vs. 51.7 years), and a different distribution of places of death (higher for public space, workplace and place of confinement and lower for health facility in cases with toxicological investigation). Conversely, substantially similar distribution for nationality, season, cadaveric state can be noted. In terms of mechanism and manner of death, finally, these directly affected the decision to implement toxicological analysis and cannot be used to compare of their representativeness. In addition, the toxicological evaluation was not performed in all cases. In judicial autopsies, they are performed only when required by the Prosecutor if toxicological data are considered helpful for legal purposes. For administrative autopsies, the toxicological assessment is performed only when needed for the identification of the cause of death. Finally, the toxicological investigations may have missed some relevant substances, although updates to the toxicological panel of xenobiotics are continuously performed based on national and international recommendations as well as developments in the licit and illicit drug markets [41–43].

## Conclusions

This retrospective study carried out in post-mortem cases in the Modena and Reggio Emilia provinces highlights that increasing age and accidental manner of death are associated with positive toxicological findings, when overall substances are considered. Benzodiazepines, alcohol and abuse drugs tend to be the most frequently detected substances, alone or in combination with both licit and illicit drugs. The most frequently detected single analyte in our study population was alcohol followed by cocaine, confirming high levels of consumption and potential abuse in the source population. Our findings also indicate a high frequency of consumption for two or more classes, in some cases up to six different classes, indicating a potential for co-exposure to these substances and a high risk of interaction between them.

This study allows to reassert that systematic toxicological investigations are relevant and valuable information that impact on epidemiological data and help establish the role of xenobiotics in the medico-legal evaluation. From a public health point of view, such an assessment can assume an important relevance for preventive policies and care strategies. This systematization provides a necessary starting point for further investigation of individual aspects of forensic interest.

## Supplementary Information

Supplementary Material 1.
