# Supplementary material for: Socio-demographic and toxicological findings from autoptic cases in a Northern Italy community (2017–2022)
Source: Int J Legal Med. 2025 Feb 3;139(3):1093–104. doi: 10.1007/s00414-025-03433-1 (PMC12003492; doi:10.1007/s00414-025-03433-1)
Supplement: Supplementary file 1 — Supplementary Material 1. [file 414_2025_3433_MOESM1_ESM.docx]

**SUPPLEMENTARY MATERIAL**

**Supplementary** **Table S1.** Limit of detection (LOD) and quantification (LOQ) of investigated xenobiotics, divided by group.

| **CATEGORIES** | **ANALYTE** | **Limit of detection (LOD)** | **Lowest limit of quantification (LLOQ)** |
| --- | --- | --- | --- |
|  |  |  |  |
| **ALCOHOL** | Ethanol | 0.01 g/L | 0.05 g/L |
| **BENZODIAZEPINES** | Alprazolam | 1 ng/mL | 5 ng/mL |
|  | α-HydroxyAlprazolam | 1 ng/mL | 5 ng/mL |
|  | Bromazepam | 5 ng/mL | 10 ng/mL |
|  | 3-HydroxyBromazepam | 5 ng/mL | 10 ng/mL |
|  | Clobazam | 1 ng/mL | 5 ng/mL |
|  | N-DesmethylClobazam | 1 ng/mL | 5 ng/mL |
|  | Clonazepam | 1 ng/mL | 5 ng/mL |
|  | 7-AminoClonazepam | 1 ng/mL | 5 ng/mL |
|  | Delorazepam | 1 ng/mL | 5 ng/mL |
|  | Diazepam | 1 ng/mL | 5 ng/mL |
|  | Nordazepam | 1 ng/mL | 5 ng/mL |
|  | Themazepam | 1 ng/mL | 5 ng/mL |
|  | Oxazepam | 1 ng/mL | 5 ng/mL |
|  | Flurazepam | 1 ng/mL | 5 ng/mL |
|  | 2-HydroxyEthylFlurazepam | 1 ng/mL | 5 ng/mL |
|  | Desalkylflurazepam | 1 ng/mL | 5 ng/mL |
|  | Lorazepam | 1 ng/mL | 5 ng/mL |
|  | Lormetazepam | 0.5 ng/mL | 2 ng/mL |
|  | Midazolam | 1 ng/mL | 5 ng/mL |
|  | 4-Hydroxymidazolam | 1 ng/mL | 5 ng/mL |
|  | Triazolam | 1 ng/mL | 5 ng/mL |
|  | α-HydroxyTriazolam | 1 ng/mL | 5 ng/mL |
|  | Zolpidem | 1 ng/mL | 5 ng/mL |
|  | Zolpidem 6-Carboxylic Acid | 1 ng/mL | 5 ng/mL |
| **CARBON MONOXIDE** | Carboxyhemoglobin | / | 10% |
| **ANTIDEPRESSANT** | Amytriptyline | 1 ng/mL | 5 ng/mL |
|  | NorTriptyline | 1 ng/mL | 5 ng/mL |
|  | Bupropion | 1 ng/mL | 5 ng/mL |
|  | Citalopram | 1 ng/mL | 5 ng/mL |
|  | N-DesmethylCitalopram | 1 ng/mL | 5 ng/mL |
|  | Duloxetine | 1 ng/mL | 5 ng/mL |
|  | Fluoxetine | 1 ng/mL | 5 ng/mL |
|  | Fluvoxamine | 1 ng/mL | 5 ng/mL |
|  | NorFluoxetine | 1 ng/mL | 5 ng/mL |
|  | Mirtazapine | 1 ng/mL | 5 ng/mL |
|  | N-DesmethylMirtazapine | 1 ng/mL | 5 ng/mL |
|  | Paroxetine | 1 ng/mL | 5 ng/mL |
|  | Reboxetin | 1 ng/mL | 5 ng/mL |
|  | Sertraline | 1 ng/mL | 5 ng/mL |
|  | DesmethylSertraline | 5 ng/mL | 10 ng/mL |
|  | Trazodone | 1 ng/mL | 5 ng/mL |
|  | Venlafaxine | 1 ng/mL | 5 ng/mL |
|  | N-DesmethylVenlafaxine | 1 ng/mL | 5 ng/mL |
|  | O-DesmethylVenlafaxine | 1 ng/mL | 5 ng/mL |
| **ANTICONVULSANT** | Carbamazepine | 1 ng/mL | 5 ng/mL |
|  | Oxcarbazepine | 1 ng/mL | 5 ng/mL |
|  | Gabapentin | 1 ng/mL | 5 ng/mL |
|  | Lacosamide | 1 ng/mL | 5 ng/mL |
|  | Lamotrigine | 1 ng/mL | 5 ng/mL |
|  | Levetiracetam | 1 ng/mL | 5 ng/mL |
|  | Phenobarbital | 5 ng/mL | 10 ng/mL |
|  | Pentobarbital | 5 ng/mL | 10 ng/mL |
|  | Pregabalin | 1 ng/mL | 5 ng/mL |
|  | Primidone | 1 ng/mL | 5 ng/mL |
|  | Valproic Acid | 10 ng/mL | 20 ng/mL |
| **DRUGS OF ABUSE** | Amphetamine | 0.5 ng/mL | 5 ng/mL |
|  | Cocaine | 0.5 ng/mL | 5 ng/mL |
|  | Benzoylecgonine | 1 ng/mL | 5 ng/mL |
|  | Cocaethylene | 0.5 ng/mL | 5 ng/mL |
|  | 3,4-methylenedioxymethamphetamine (MDMA) | 0.5 ng/mL | 5 ng/mL |
|  | 3,4-Methylenedioxyamphetamine (MDA) | 0.5 ng/mL | 5 ng/mL |
|  | 6-MonoAcetylMorphine | 0.5 ng/mL | 5 ng/mL |
|  | 6-DesmethylPapaverine | 0.1 ng/mL | 5 ng/mL |
|  | Mephedrone | 1 ng/mL | 5 ng/mL |
|  | NorMephedrone | 1 ng/mL | 5 ng/mL |
|  | delta-9-tetrahydrocannabinol | 0.1 ng/mL | 2 ng/mL |
|  | 11-Hydroxy-delta-9-tetrahydrocannabinol | 0.2 ng/mL | 1 ng/mL |
|  | 11-Nor-9-carboxy-delta-9-tetrahydrocannabinol | 1 ng/mL | 5 ng/mL |
|  | 11-Nor-9-carboxy-delta-9-tetrahydrocannabinol glucuronide | 1 ng/mL | 5 ng/mL |
| **OPIOID AGONIST** | Buprenorphine | 1 ng/mL | 5 ng/mL |
|  | NorBuprenorphine | 1 ng/mL | 5 ng/mL |
|  | Codeine | 0.5 ng/mL | 5 ng/mL |
|  | NorCodeine | 0.5 ng/mL | 5 ng/mL |
|  | Dihydrocodeine | 0.5 ng/mL | 5 ng/mL |
|  | Fentanyl | 0.05 ng/mL | 1 ng/mL |
|  | Norfentanyl | 0.05 ng/mL | 1 ng/mL |
|  | Methadone | 1 ng/mL | 5 ng/mL |
|  | 2-ethylidene-1,5 dimethyl-3,3-diphenyl-pyrrolidine (EDDP) | 1 ng/mL | 5 ng/mL |
|  | Morphine | 0.5 ng/mL | 5 ng/mL |
|  | Oxycodone | 1 ng/mL | 5 ng/mL |
|  | NorOxycodone | 1 ng/mL | 5 ng/mL |
|  | Oxymorphone | 1 ng/mL | 5 ng/mL |
|  | Tramadol | 1 ng/mL | 10 ng/mL |
|  | N-DesmethylTramadol | 1 ng/mL | 10 ng/mL |
|  | O-DesmethylTramadol | 1 ng/mL | 10 ng/mL |
|  | N-desmethylTapentadol | 1 ng/mL | 10 ng/mL |
| **ANTIPSYCHOTICS** | Amisulpiride | 1 ng/mL | 10 ng/mL |
|  | Aripiprazole | 1 ng/mL | 10 ng/mL |
|  | Clotiapine | 1 ng/mL | 10 ng/mL |
|  | Clozapine | 1 ng/mL | 10 ng/mL |
|  | N-DesmethylClozapine | 1 ng/mL | 10 ng/mL |
|  | Haloperidol | 1 ng/mL | 10 ng/mL |
|  | Haloperidol metabolite | 1 ng/mL | 10 ng/mL |
|  | Levomepromazine | 1 ng/mL | 10 ng/mL |
|  | Olanzapine | 1 ng/mL | 10 ng/mL |
|  | Levosulpiride | 1 ng/mL | 10 ng/mL |
|  | Promazine | 5 ng/mL | 10 ng/mL |
|  | Quetiapine | 1 ng/mL | 5 ng/mL |
|  | NorQuetiapine | 1 ng/mL | 5 ng/mL |
|  | 7-HydroxyQuetiapine | 5 ng/mL | 10 ng/mL |
|  | Paliperidone | 1 ng/mL | 5 ng/mL |
|  | Risperidone | 1 ng/mL | 5 ng/mL |
|  | Sulpiride | 1 ng/mL | 5 ng/mL |
|  | Tiapride | 1 ng/mL | 5 ng/mL |
| **ANTIHYPERTENSIVES** | Amlodipine | 1 ng/mL | 5 ng/mL |
|  | Atenolol | 1 ng/mL | 5 ng/mL |
|  | Bisoprolol | 1 ng/mL | 5 ng/mL |
|  | Carvedilol | 5 ng/mL | 10 ng/mL |
|  | 4-HydroxyCarvedilol | 5 ng/mL | 10 ng/mL |
|  | Diltiazem | 1 ng/mL | 5 ng/mL |
|  | Doxazosin | 1 ng/mL | 5 ng/mL |
|  | Hydrochlorothiazide | 1 ng/mL | 5 ng/mL |
|  | Metoprolol | 1 ng/mL | 5 ng/mL |
|  | Ramipril | 1 ng/mL | 5 ng/mL |
|  | Valsartan | 1 ng/mL | 5 ng/mL |
| **OTHER** | Acetone | 0.01 g/L | 0.05 g/L |
|  | Aliphatic Hydrocarbons (butane) | 5 ng/mL | 50 ng/mL |
|  | Biperiden | 1 ng/mL | 5 ng/mL |
|  | Chlorpheniramine | 1 ng/mL | 5 ng/mL |
|  | Ephedrine | 1 ng/mL | 5 ng/mL |
|  | NorEphedrine | 1 ng/mL | 5 ng/mL |
|  | PseudoEphedrine | 1 ng/mL | 5 ng/mL |
|  | PseudoNorEphedrine | 1 ng/mL | 5 ng/mL |
|  | Hydroxyzine | 1 ng/mL | 5 ng/mL |
|  | Isopropanol | 0.002 g/L | 0.02 g/L |
|  | Ketamine | 1 ng/mL | 5 ng/mL |
|  | NorKetamine | 1 ng/mL | 5 ng/mL |
|  | Lidocaine | 1 ng/mL | 5 ng/mL |
|  | Metformin | 10 ng/mL | 25 ng/mL |
|  | Metoclopramide | 1 ng/mL | 5 ng/mL |
|  | Naloxone | 1 ng/mL | 5 ng/mL |
|  | Potassium | 2 mEq/L | 3.5 mEq/L |
|  | Propofol | 20 ng/mL | 40 ng/mL |
|  | Salbutamol | 1 ng/mL | 5 ng/mL |
|  | Sildenafil | 1 ng/mL | 5 ng/mL |
|  | Ticlopidine | 1 ng/mL | 5 ng/mL |

**Supplementary** **Table S2.** Frequency of xenobiotics investigated in the study population, with positivity in total investigated cases (N=504) and in positive cases only (N=330).

| **Categories** | **Analyte** | **Number of cases in which the analyte was detected** | **Positivity on  total cases (504)** | **Positivity on  positive cases (330)** |
| --- | --- | --- | --- | --- |
| ALCOHOL | Ethanol | 117 | 23.2% | 35.5% |
| DRUGS OF ABUSE | Benzoylecgonine | 60 | 11.9% | 18.2% |
| DRUGS OF ABUSE | Cocaine | 54 | 10.7% | 16.4% |
| BENZODIAZEPINES | Lorazepam | 54 | 10.7% | 16.4% |
| DRUGS OF ABUSE | Morphine | 38 | 7.5% | 11.5% |
| DRUGS OF ABUSE | Cocaethylene | 34 | 6.7% | 10.3% |
| BENZODIAZEPINES | Diazepam | 34 | 6.7% | 10.3% |
| BENZODIAZEPINES | Nordazepam | 34 | 6.7% | 10.3% |
| DRUGS OF ABUSE | 6-MonoAcetylMorphine | 31 | 6.2% | 9.4% |
| BENZODIAZEPINES | Oxazepam | 29 | 5.8% | 8.8% |
| BENZODIAZEPINES | Alprazolam | 25 | 5.0% | 7.6% |
| OPIOID AGONIST | Methadone | 24 | 4.8% | 7.3% |
| ANTIPSYCHOTICS | Quetiapine | 24 | 4.8% | 7.3% |
| OPIOID AGONIST | 2-ethylidene-1,5 dimethyl-3,3-diphenyl-pyrrolidine (EDDP) | 23 | 4.6% | 7.0% |
| DRUGS OF ABUSE | 11-Nor-9-carboxy-delta-9-tetrahydrocannabinol | 22 | 4.4% | 6.7% |
| BENZODIAZEPINES | α-HydroxyAlprazolam | 21 | 4.2% | 6.4% |
| DRUGS OF ABUSE | delta-9-tetrahydrocannabinol | 19 | 3.8% | 5.8% |
| BENZODIAZEPINES | Delorazepam | 18 | 3.6% | 5.5% |
| ANTIHYPERTENSIVES | Amlodipine | 16 | 3.2% | 4.8% |
| OTHER | Metformin | 16 | 3.2% | 4.8% |
| CARBON MONOXIDE | Carboxyhemoglobin | 14 | 2.8% | 4.2% |
| DRUGS OF ABUSE | 11-Hydroxy-delta-9-tetrahydrocannabinol | 16 | 3.2% | 4.8% |
| ANTICONVULSANT | Pregabalin | 15 | 3.0% | 4.5% |
| ANTIDEPRESSANT | Trazodone | 15 | 3.0% | 4.5% |
| BENZODIAZEPINES | Midazolam | 14 | 2.8% | 4.2% |
| BENZODIAZEPINES | 7-AminoClonazepam | 14 | 2.8% | 4.2% |
| ANTIHYPERTENSIVES | Bisoprolol | 13 | 2.6% | 3.9% |
| ANTIDEPRESSANT | Citalopram | 13 | 2.6% | 3.9% |
| ANTIDEPRESSANT | N-DesmethylCitalopram | 12 | 2.4% | 3.6% |
| BENZODIAZEPINES | 4-Hydroxymidazolam | 12 | 2.4% | 3.6% |
| ANTIDEPRESSANT | Paroxetine | 12 | 2.4% | 3.6% |
| BENZODIAZEPINES | Clonazepam | 11 | 2.2% | 3.3% |
| BENZODIAZEPINES | α-HydroxyTriazolam | 11 | 2.2% | 3.3% |
| OTHER | Acetone | 10 | 2.0% | 3.0% |
| ANTIPSYCHOTICS | Olanzapine | 10 | 2.0% | 3.0% |
| ANTIPSYCHOTICS | Paliperidone | 10 | 2.0% | 3.0% |
| ANTIDEPRESSANT | Sertraline | 10 | 2.0% | 3.0% |
| BENZODIAZEPINES | Themazepam | 10 | 2.0% | 3.0% |
| BENZODIAZEPINES | Triazolam | 10 | 2.0% | 3.0% |
| BENZODIAZEPINES | Zolpidem 6-Carboxylic Acid | 10 | 2.0% | 3.0% |
| DRUGS OF ABUSE | 6-DesmethylPapaverine | 9 | 1.8% | 2.7% |
| OTHER | Metoclopramide | 9 | 1.8% | 2.7% |
| BENZODIAZEPINES | Zolpidem | 9 | 1.8% | 2.7% |
| OPIOID AGONIST | Codeine | 8 | 1.6% | 2.4% |
| ANTIPSYCHOTICS | Levosulpiride | 8 | 1.6% | 2.4% |
| ANTIPSYCHOTICS | NorQuetiapine | 8 | 1.6% | 2.4% |
| ANTIPSYCHOTICS | Risperidone | 8 | 1.6% | 2.4% |
| ANTIDEPRESSANT | Venlafaxine | 8 | 1.6% | 2.4% |
| ANTIHYPERTENSIVES | Atenolol | 7 | 1.4% | 2.1% |
| OTHER | Chlorpheniramine | 7 | 1.4% | 2.1% |
| ANTIDEPRESSANT | O-DesmethylVenlafaxine | 7 | 1.4% | 2.1% |
| OTHER | Salbutamol | 7 | 1.4% | 2.1% |
| ANTIPSYCHOTICS | 7-HydroxyQuetiapine | 6 | 1.2% | 1.8% |
| ANTICONVULSANT | Valproic Acid | 6 | 1.2% | 1.8% |
| BENZODIAZEPINES | Desalkylflurazepam | 6 | 1.2% | 1.8% |
| BENZODIAZEPINES | Lormetazepam | 6 | 1.2% | 1.8% |
| OPIOID AGONIST | Oxycodone | 6 | 1.2% | 1.8% |
| OPIOID AGONIST | Morphine | 5 | 1.0% | 1.5% |
| ANTICONVULSANT | Phenobarbital | 5 | 1.0% | 1.5% |
| ANTIDEPRESSANT | Mirtazapine | 5 | 1.0% | 1.5% |
| OPIOID AGONIST | Oxymorphone | 5 | 1.0% | 1.5% |
| ANTIPSYCHOTICS | Promazine | 5 | 1.0% | 1.5% |
| OPIOID AGONIST | Fentanyl | 4 | 0.8% | 1.2% |
| BENZODIAZEPINES | Flurazepam | 4 | 0.8% | 1.2% |
| ANTICONVULSANT | Gabapentin | 4 | 0.8% | 1.2% |
| ANTIDEPRESSANT | N-DesmethylMirtazapine | 4 | 0.8% | 1.2% |
| OPIOID AGONIST | NorOxycodone | 4 | 0.8% | 1.2% |
| ANTIDEPRESSANT | DesmethylSertraline | 4 | 0.8% | 1.2% |
| BENZODIAZEPINES | 2-HydroxyEthylFlurazepam | 4 | 0.8% | 1.2% |
| DRUGS OF ABUSE | 11-Nor-9-carboxy-delta-9-tetrahydrocannabinol glucuronide | 4 | 0.8% | 1.2% |
| OPIOID AGONIST | Tramadol | 4 | 0.8% | 1.2% |
| ANTIHYPERTENSIVES | Valsartan | 4 | 0.8% | 1.2% |
| ANTIPSYCHOTICS | Haloperidol | 3 | 0.6% | 0.9% |
| OTHER | Biperiden | 3 | 0.6% | 0.9% |
| BENZODIAZEPINES | Bromazepam | 3 | 0.6% | 0.9% |
| OPIOID AGONIST | Buprenorphine | 3 | 0.6% | 0.9% |
| ANTIDEPRESSANT | Duloxetine | 3 | 0.6% | 0.9% |
| OTHER | Ketamine | 3 | 0.6% | 0.9% |
| ANTICONVULSANT | Lamotrigine | 3 | 0.6% | 0.9% |
| ANTIPSYCHOTICS | Levomepromazine | 3 | 0.6% | 0.9% |
| OPIOID AGONIST | N-DesmethylTramadol | 3 | 0.6% | 0.9% |
| OPIOID AGONIST | NorBuprenorphine | 3 | 0.6% | 0.9% |
| ANTIDEPRESSANT | NorTriptyline | 3 | 0.6% | 0.9% |
| OPIOID AGONIST | O-DesmethylTramadol | 3 | 0.6% | 0.9% |
| BENZODIAZEPINES | 3-HydroxyBromazepam | 3 | 0.6% | 0.9% |
| ANTICONVULSANT | Oxcarbazepine | 3 | 0.6% | 0.9% |
| OTHER | Sildenafil | 3 | 0.6% | 0.9% |
| ANTIPSYCHOTICS | Haloperidol metabolite | 2 | 0.4% | 0.6% |
| ANTIDEPRESSANT | Amytriptyline | 2 | 0.4% | 0.6% |
| ANTIDEPRESSANT | Bupropion | 2 | 0.4% | 0.6% |
| ANTICONVULSANT | Carbamazepine | 2 | 0.4% | 0.6% |
| ANTIHYPERTENSIVES | Carvedilol | 2 | 0.4% | 0.6% |
| BENZODIAZEPINES | Clobazam | 2 | 0.4% | 0.6% |
| ANTIPSYCHOTICS | Clotiapine | 2 | 0.4% | 0.6% |
| ANTIHYPERTENSIVES | Diltiazem | 2 | 0.4% | 0.6% |
| ANTIHYPERTENSIVES | Doxazosin | 2 | 0.4% | 0.6% |
| ANTIDEPRESSANT | Fluvoxamine | 2 | 0.4% | 0.6% |
| OTHER | Aliphatic Hydrocarbons (butane) | 2 | 0.4% | 0.6% |
| OTHER | Lidocaine | 2 | 0.4% | 0.6% |
| DRUGS OF ABUSE | MDA (3,4-Methylenedioxyamphetamine) | 2 | 0.4% | 0.6% |
| DRUGS OF ABUSE | MDMA (3,4-methylenedioxymethamphetamine) | 2 | 0.4% | 0.6% |
| ANTIHYPERTENSIVES | Metoprolol | 2 | 0.4% | 0.6% |
| BENZODIAZEPINES | N-DesmethylClobazam | 2 | 0.4% | 0.6% |
| OPIOID AGONIST | Norfentanyl | 2 | 0.4% | 0.6% |
| OTHER | NorKetamine | 2 | 0.4% | 0.6% |
| OTHER | PseudoNorEphedrine | 2 | 0.4% | 0.6% |
| ANTIDEPRESSANT | N-DesmethylVenlafaxine | 2 | 0.4% | 0.6% |
| OTHER | PseudoEphedrine | 2 | 0.4% | 0.6% |
| DRUGS OF ABUSE | Amphetamine | 1 | 0.2% | 0.3% |
| ANTIPSYCHOTICS | Amisulpiride | 1 | 0.2% | 0.3% |
| ANTIPSYCHOTICS | Aripiprazole | 1 | 0.2% | 0.3% |
| ANTIPSYCHOTICS | Clozapine | 1 | 0.2% | 0.3% |
| ANTIDEPRESSANT | N-DesmethylCitalopram | 1 | 0.2% | 0.3% |
| OPIOID AGONIST | Dihydrocodeine | 1 | 0.2% | 0.3% |
| OTHER | Ephedrine | 1 | 0.2% | 0.3% |
| ANTIDEPRESSANT | Fluoxetine | 1 | 0.2% | 0.3% |
| ANTIHYPERTENSIVES | Hydrochlorothiazide | 1 | 0.2% | 0.3% |
| OTHER | Isopropanol | 1 | 0.2% | 0.3% |
| ANTICONVULSANT | Lacosamide | 1 | 0.2% | 0.3% |
| ANTICONVULSANT | Levetiracetam | 1 | 0.2% | 0.3% |
| DRUGS OF ABUSE | Mephedrone | 1 | 0.2% | 0.3% |
| OTHER | Naloxone | 1 | 0.2% | 0.3% |
| ANTIPSYCHOTICS | N-DesmethylClozapine | 1 | 0.2% | 0.3% |
| OPIOID AGONIST | N-DesmethylTapentadol | 1 | 0.2% | 0.3% |
| OPIOID AGONIST | NorCodeine | 1 | 0.2% | 0.3% |
| OTHER | NorEphedrine | 1 | 0.2% | 0.3% |
| ANTIDEPRESSANT | NorFluoxetine | 1 | 0.2% | 0.3% |
| DRUGS OF ABUSE | Normephedrone | 1 | 0.2% | 0.3% |
| ANTIHYPERTENSIVES | 4-HydroxyCarvedilol | 1 | 0.2% | 0.3% |
| ANTICONVULSANT | Pentobarbital | 1 | 0.2% | 0.3% |
| OTHER | Potassium | 1 | 0.2% | 0.3% |
| ANTICONVULSANT | Primidone | 1 | 0.2% | 0.3% |
| OTHER | Propofol | 1 | 0.2% | 0.3% |
| ANTIHYPERTENSIVES | Ramipril | 1 | 0.2% | 0.3% |
| ANTIDEPRESSANT | Reboxetin | 1 | 0.2% | 0.3% |
| ANTIPSYCHOTICS | Sulpiride | 1 | 0.2% | 0.3% |
| OTHER | Ticlopidine | 1 | 0.2% | 0.3% |
| ANTIPSYCHOTICS | Tiapride | 1 | 0.2% | 0.3% |
| OTHER | Hydroxyzine | 1 | 0.2% | 0.3% |

**Supplementary** **Table S3.** Odds ratio (OR) with 95% confidence interval for demographic and circumstantial characteristics.

|  |  |  | ***Crude*** |  |  | ***Adjusted*** |  |
| --- | --- | --- | --- | --- | --- | --- | --- |
| Drug abuse  n=82/248 | ***Y/N*** | ***OR*** | ***95% CI*** | ***P value*** | ***OR*** | ***95% CI*** | ***P value*** |
| ***Sex*** |  |  |  |  |  |  |  |
| Male | 73/161 | 1.00 | - |  | 1.00 | - |  |
| Female | 9/87 | 0.23 | (0.11-0.48) | <0.001 | 0.31 | (0.14-0.70) | 0.005 |
| ***Age*** |  |  |  |  |  |  |  |
| ≤ 30 years | 12/14 | 1.00 | - |  | 1.00 | - |  |
| > 30 ≤ 60 years | 67/139 | 0.56 | (0.25-1.28) | 0.171 | 0.64 | (0.25-1.61) | 0.339 |
| > 60 years | 3/95 | 0.04 | (0.01-0.15) | <0.001 | 0.05 | (0.01-0.21) | <0.001 |
| ***Nationality*** |  |  |  |  |  |  |  |
| Italians | 62/178 | 1.00 | - |  | 1.00 | - |  |
| Non-Italians | 20/70 | 0.82 | (0.46-1.46) | 0.499 | 0.54 | (0.28-1.07) | 0.077 |
| ***Place of death*** |  |  |  |  |  |  |  |
| House | 52/157 | 1.00 | - |  | 1.00 | - |  |
| Public space | 20/53 | 1.14 | (0.62-2.08) | 0.671 | 0.70 | (0.33-1.46) | 0.337 |
| Health facility | 4/9 | 0.64 | (0.21-1.95) | 0.429 | 0.49 | (0.14-1.75) | 0.274 |
| Workplace | 3/10 | 0.91 | (0.24-3.42) | 0.884 | 0.44 | (0.11-1.85) | 0.265 |
| Place of confinement | 3/9 | 1.01 | (0.26-3.86) | 0.993 | 0.48 | (0.11-2.09) | 0.326 |
| ***Season*** |  |  |  |  |  |  |  |
| Spring | 18/65 | 1.00 | - |  | 1.00 | - |  |
| Summer | 27/68 | 1.43 | (0.72-2.85) | 0.304 | 1.58 | (0.72-3.48) | 0.252 |
| Autumn | 16/49 | 1.18 | (0.55-2.54) | 0.674 | 1.17 | (0.48-2.85) | 0.730 |
| Winter | 21/66 | 1.15 | (0.56-2.35) | 0.704 | 1.15 | (0.51-2.61) | 0.734 |
| ***Manner of death*** |  |  |  |  |  |  |  |
| Natural Cause | 34/136 | 1.00 | - |  | 1.00 | - |  |
| Accidental | 44/68 | 2.59 | (1.52-4.41) | <0.001 | 2.82 | (1.49-5.33) | 0.001 |
| Suicide | 2/33 | 0.24 | (0.06-1.06) | 0.060 | 0.29 | (0.06-1.37) | 0.118 |
| Homicide | 2/11 | 0.73 | (0.15-3.44) | 0.668 | 1.42 | (0.24-8.32) | 0.695 |

Adjusted for sex, age category, nationality, place of death, season, and manner of death.

**Supplementary** **Table S4.** Odds ratio (OR) with 95% confidence interval for demographic and circumstantial characteristics.

|  |  |  | ***Crude*** |  |  | ***Adjusted*** |  |
| --- | --- | --- | --- | --- | --- | --- | --- |
| Alcohol  n=117/213 | ***Y/N*** | ***OR*** | ***95% CI*** | ***P value*** | ***OR*** | ***95% CI*** | ***P value*** |
| ***Sex*** |  |  |  |  |  |  |  |
| Male | 89/145 | 1.00 | - |  | 1.00 | - |  |
| Female | 28/68 | 0.67 | (0.40-1.12) | 0.127 | 0.78 | (0.44-1.37) | 0.385 |
| ***Age*** |  |  |  |  |  |  |  |
| ≤ 30 years | 11/15 | 1.00 | - |  | 1.00 | - |  |
| > 30 ≤ 60 years | 81/125 | 0.88 | (0.39-2.02) | 0.769 | 0.86 | (0.35-2.10) | 0.743 |
| > 60 years | 25/73 | 0.47 | (0.19-1.16) | 0.098 | 0.46 | (0.17-1.23) | 0.122 |
| ***Nationality*** |  |  |  |  |  |  |  |
| Italians | 79/161 | 1.00 | - |  | 1.00 | - |  |
| Non-Italians | 38/52 | 1.49 | (0.91-2.45) | 0.117 | 1.64 | (0.95-2.81) | 0.075 |
| ***Place of death*** |  |  |  |  |  |  |  |
| House | 73/136 | 1.00 | - |  | 1.00 | - |  |
| Public space | 34/39 | 1.67 | (0.95-2.79) | 0.079 | 1.56 | (0.86-2.80) | 0.141 |
| Health facility | 4/19 | 0.39 | (0.13-1.20) | 0.100 | 0.35 | (0.11-1.12) | 0.076 |
| Workplace | 4/9 | 0.83 | (0.25-2.78) | 0.760 | 0.61 | (0.17-2.16) | 0.445 |
| Place of confinement | 2/10 | 0.37 | (0.08-1.75) | 0.210 | 0.21 | (0.04-1.08) | 0.062 |
| ***Season*** |  |  |  |  |  |  |  |
| Spring | 26/57 | 1.00 | - |  | 1.00 | - |  |
| Summer | 41/54 | 1.66 | (0.90-3.08) | 0.105 | 1.86 | (0.98-3.55) | 0.060 |
| Autumn | 19/46 | 0.91 | (0.45-1.84) | 0.783 | 0.86 | (0.41-1.81) | 0.684 |
| Winter | 31/56 | 1.21 | (0.64-2.30) | 0.552 | 1.26 | (0.64-2.47) | 0.501 |
| ***Manner of death*** |  |  |  |  |  |  |  |
| Natural Cause | 58/112 | 1.00 | - |  | 1.00 | - |  |
| Accidental | 42/70 | 1.16 | (0.70-1.90) | 0.561 | 1.04 | (0.60-1.81) | 0.877 |
| Suicide | 13/22 | 1.14 | (0.54-2.43) | 0.732 | 1.20 | (0.54-2.66) | 0.660 |
| Homicide | 4/9 | 0.86 | (0.25-2.91) | 0.806 | 0.87 | (0.23-3.25) | 0.839 |

Adjusted for sex, age category, nationality, place of death, season, and manner of death.

**Supplementary** **Table S5.** Odds ratio (OR) with 95% confidence interval for demographic and circumstantial characteristics.

|  |  |  | ***Crude*** |  |  | ***Adjusted*** |  |
| --- | --- | --- | --- | --- | --- | --- | --- |
| Benzodiazepines  n=136/194 | ***Y/N*** | ***OR*** | ***95% CI*** | ***P value*** | ***OR*** | ***95% CI*** | ***P value*** |
| ***Sex*** |  |  |  |  |  |  |  |
| Male | 83/151 | 1.00 | - |  | 1.00 | - |  |
| Female | 53/43 | 2.24 | (1.38-3.64) | 0.001 | 2.66 | (1.51-4.69) | 0.001 |
| ***Age*** |  |  |  |  |  |  |  |
| ≤ 30 years | 8/18 | 1.00 | - |  | 1.00 | - |  |
| > 30 ≤ 60 years | 90/116 | 1.75 | (0.73-4.20) | 0.213 | 2.91 | (1.04-8.15) | 0.042 |
| > 60 years | 38/60 | 1.42 | (0.56-3.60) | 0.454 | 1.42 | (0.48-4.24) | 0.526 |
| ***Nationality*** |  |  |  |  |  |  |  |
| Italians | 107/133 | 1.00 | - |  | 1.00 | - |  |
| Non-Italians | 29/61 | 0.59 | (0.35-0.98) | 0.043 | 0.40 | (0.22-0.75) | 0.004 |
| ***Place of death*** |  |  |  |  |  |  |  |
| House | 90/119 | 1.00 | - |  | 1.00 | - |  |
| Public space | 21/52 | 0.53 | (0.30-0.95) | 0.033 | 0.56 | (0.29-1.06) | 0.078 |
| Health facility | 13/10 | 1.72 | (0.72-4.10) | 0.222 | 2.29 | (0.86-5.85) | 0.085 |
| Workplace | 1/12 | 0.11 | (0.01-0.86) | 0.036 | 0.16 | (0.02-1.29) | 0.086 |
| Place of confinement | 11/1 | 14.54 | (1.84-114.73) | 0.011 | 33.18 | (3.80-289.60) | 0.002 |
| ***Season*** |  |  |  |  |  |  |  |
| Spring | 34/49 | 1.00 | - |  | 1.00 | - |  |
| Summer | 41/54 | 1.09 | (0.60-1.99) | 0.767 | 1.09 | (0.55-2.16) | 0.796 |
| Autumn | 31/34 | 1.31 | (0.68-2.53) | 0.413 | 1.93 | (0.91-4.07) | 0.085 |
| Winter | 30/57 | 0.76 | (0.41-1.41) | 0.384 | 0.80 | (0.39-1.64) | 0.543 |
| ***Manner of death*** |  |  |  |  |  |  |  |
| Natural Cause | 59/111 | 1.00 | - |  | 1.00 | - |  |
| Accidental | 48/64 | 1.41 | (0.86-2.30) | 0.168 | 1.96 | (1.09-3.52) | 0.024 |
| Suicide | 24/11 | 4.10 | (1.88-8.96) | <0.001 | 5.66 | (2.38-13.44) | <0.001 |
| Homicide | 5/8 | 1.18 | (0.37-3.76) | 0.785 | 1.06 | (0.30-3.71) | 0.929 |

Adjusted for sex, age category, nationality, place of death, season, and manner of death.

**Supplementary** **Table S6.** Odds ratio (OR) with 95% confidence interval for demographic and circumstantial characteristics.

|  |  |  | ***Crude*** |  |  | ***Adjusted*** |  |
| --- | --- | --- | --- | --- | --- | --- | --- |
| Antidepressants  n=59/271 | ***Y/N*** | ***OR*** | ***95% CI*** | ***P value*** | ***OR*** | ***95% CI*** | ***P value*** |
| ***Sex*** |  |  |  |  |  |  |  |
| Male | 33/201 | 1.00 | - |  | 1.00 | - |  |
| Female | 26/70 | 2.26 | (1.26-4.05) | 0.006 | 2.26 | (1.17-4.38) | 0.015 |
| ***Age*** |  |  |  |  |  |  |  |
| ≤ 30 years | 2/24 | 1.00 | - |  | 1.00 | - |  |
| > 30 ≤ 60 years | 35/171 | 2.46 | (0.55-10.87) | 0.236 | 2.68 | (0.57-12.72) | 0.214 |
| > 60 years | 22/76 | 3.47 | (0.76-15.86) | 0.108 | 2.58 | (0.52-12.76) | 0.245 |
| ***Nationality*** |  |  |  |  |  |  |  |
| Italians | 56/184 | 1.00 | - |  | 1.00 | - |  |
| Non-Italians | 3/87 | 0.11 | (0.03-0.37) | <0.001 | 0.08 | (0.02-0.31) | <0.001 |
| ***Place of death*** |  |  |  |  |  |  |  |
| House | 43/166 | 1.00 | - |  | 1.00 | - |  |
| Public space | 10/63 | 0.61 | (0.29-1.29) | 0.199 | 0.78 | (0.34-1.76) | 0.547 |
| Health facility | 3/20 | 0.58 | (0.16-2.04) | 0.395 | 0.65 | (0.17-2.48) | 0.532 |
| Workplace | 0/13 | - | - |  | - | - |  |
| Place of confinement | 3/9 | 1.29 | (0.33-4.96) | 0.714 | 7.58 | (1.28-44.74) | 0.025 |
| ***Season*** |  |  |  |  |  |  |  |
| Spring | 13/70 | 1.00 | - |  | 1.00 | - |  |
| Summer | 20/75 | 1.44 | (0.66-3.10) | 0.357 | 1.26 | (0.55-2.88) | 0.590 |
| Autumn | 14/51 | 1.48 | (0.64-3.41) | 0.360 | 1.50 | (0.60-3.75) | 0.385 |
| Winter | 12/75 | 0.86 | (0.37-2.01) | 0.731 | 0.79 | (0.31-1.97) | 0.610 |
| ***Manner of death*** |  |  |  |  |  |  |  |
| Natural Cause | 30/140 | 1.00 | - |  | 1.00 | - |  |
| Accidental | 18/94 | 0.89 | (0.47-1.69) | 0.731 | 1.25 | (0.61-2.55) | 0.539 |
| Suicide | 9/26 | 1.62 | (0.69-3.80) | 0.271 | 1.60 | (0.63-4.04) | 0.323 |
| Homicide | 2/11 | 0.85 | (0.18-4.03) | 0.836 | 0.72 | (0.14-3.80) | 0.698 |

Adjusted for sex, age category, nationality, place of death, season, and manner of death.

**Supplementary** **Table S7.** Odds ratio (OR) with 95% confidence interval for demographic and circumstantial characteristics.

|  |  |  | ***Crude*** |  |  | ***Adjusted*** |  |
| --- | --- | --- | --- | --- | --- | --- | --- |
| Antipsychotics  n=63/267 | ***Y/N*** | ***OR*** | ***95% CI*** | ***P value*** | ***OR*** | ***95% CI*** | ***P value*** |
| ***Sex*** |  |  |  |  |  |  |  |
| Male | 45/189 | 1.00 | - |  | 1.00 | - |  |
| Female | 18/78 | 0.97 | (0.53-1.78) | 0.920 | 1.07 | (0.54-2.13) | 0.844 |
| ***Age*** |  |  |  |  |  |  |  |
| ≤ 30 years | 5/21 | 1.00 | - |  | 1.00 | - |  |
| > 30 ≤ 60 years | 44/162 | 1.14 | (0.41-3.20) | 0.802 | 1.29 | (0.42-4.02) | 0.657 |
| > 60 years | 14/84 | 0.70 | (0.23-2.16) | 0.535 | 0.68 | (0.19-2.37) | 0.542 |
| ***Nationality*** |  |  |  |  |  |  |  |
| Italians | 55/185 | 1.00 | - |  | 1.00 | - |  |
| Non-Italians | 8/82 | 0.33 | (0.15-0.82) | 0.005 | 0.19 | (0.07-0.52) | 0.001 |
| ***Place of death*** |  |  |  |  |  |  |  |
| House | 46/143 | 1.00 | - |  | 1.00 | - |  |
| Public space | 6/67 | 0.32 | (0.13-0.78) | 0.012 | 0.30 | (0.12-0.78) | 0.014 |
| Health facility | 5/18 | 0.98 | (0.35-2.79) | 0.976 | 1.22 | (0.40-3.76) | 0.727 |
| Workplace | 0/13 | - | - |  | - | - |  |
| Place of confinement | 6/6 | 3.54 | (1.09-11.51) | 0.035 | 8.33 | (1.88-36.91) | 0.005 |
| ***Season*** |  |  |  |  |  |  |  |
| Spring | 13/70 | 1.00 | - |  | 1.00 | - |  |
| Summer | 25/70 | 1.92 | (0.91-4.06) | 0.086 | 2.04 | (0.89-4.68) | 0.091 |
| Autumn | 12/53 | 1.22 | (0.51-2.89) | 0.652 | 1.57 | (0.60-4.13) | 0.359 |
| Winter | 13/74 | 0.95 | (0.41-2.18) | 0.896 | 0.932 | (0.37-2.33) | 0.873 |
| ***Manner of death*** |  |  |  |  |  |  |  |
| Natural Cause | 28/142 | 1.00 | - |  | 1.00 | - |  |
| Accidental | 24/88 | 1.38 | (0.75-2.54) | 0.295 | 1.97 | (0.99-3.91) | 0.056 |
| Suicide | 11/24 | 2.32 | (1.02-5.28) | 0.044 | 2.93 | (1.19-7.22) | 0.019 |
| Homicide | 0/13 | - | - |  | - | - |  |

Adjusted for sex, age category, nationality, place of death, season, and manner of death.

**Supplementary** **Table S8.** Odds ratio (OR) with 95% confidence interval for demographic and circumstantial characteristics.

|  |  |  | ***Crude*** |  |  | ***Adjusted*** |  |
| --- | --- | --- | --- | --- | --- | --- | --- |
| Anticonvulsants  n=32/298 | ***Y/N*** | ***OR*** | ***95% CI*** | ***P value*** | ***OR*** | ***95% CI*** | ***P value*** |
| ***Sex*** |  |  |  |  |  |  |  |
| Male | 21/213 | 1.00 | - |  | 1.00 | - |  |
| Female | 11/85 | 1.31 | (0.61-2.84) | 0.490 | 1.33 | (0.55-3.21) | 0.524 |
| ***Age*** |  |  |  |  |  |  |  |
| ≤ 30 years | 1/25 | 1.00 | - |  | 1.00 | - |  |
| > 30 ≤ 60 years | 24/182 | 3.30 | (0.43-25.44) | 0.253 | 3.93 | (0.44-35.38) | 0.222 |
| > 60 years | 7/91 | 1.92 | (0.23-16.37) | 0.550 | 2.16 | (0.21-22.23) | 0.519 |
| ***Nationality*** |  |  |  |  |  |  |  |
| Italians | 22/218 | 1.00 | - |  | 1.00 | - |  |
| Non-Italians | 10/80 | 1.24 | (0.56-2.73) | 0.596 | 0.86 | (0.33-2.27) | 0.759 |
| ***Place of death*** |  |  |  |  |  |  |  |
| House | 23/186 | 1.00 | - |  | 1.00 | - |  |
| Public space | 0/73 | - |  |  | - | - |  |
| Health facility | 4/19 | 1.70 | (0.53-5.44) | 0.950 | 2.27 | (0.64-8.07) | 0.205 |
| Workplace | 0/13 | - |  |  | - | - |  |
| Place of confinement | 5/7 | 7.78 | (1.69-19.70) | 0.002 | 5.69 | (1.27-25.51) | 0.023 |
| ***Season*** |  |  |  |  |  |  |  |
| Spring | 13/70 | 1.00 | - |  | 1.00 | - |  |
| Summer | 11/84 | 0.71 | (0.30-1.28) | 0.428 | 0.73 | (0.28-1.89) | 0.511 |
| Autumn | 6/59 | 0.55 | (0.20-1.53) | 0.251 | 0.80 | (0.26-2.48) | 0.703 |
| Winter | 2/85 | 0.13 | (0.03-0.59) | 0.008 | 0.13 | (0.03-0.66) | 0.014 |
| ***Manner of death*** |  |  |  |  |  |  |  |
| Natural Cause | 14/156 | 1.00 | - |  | 1.00 | - |  |
| Accidental | 1/101 | 1.21 | (0.53-2.78) | 0.760 | 1.55 | (0.61-3.90) | 0.356 |
| Suicide | 5/30 | 1.86 | (0.62-5.54) | 0.385 | 2.83 | (0.85-9.41) | 0.089 |
| Homicide | 2/11 | 2.03 | (0.41-10.06) | 0.932 | 2.05 | (0.36-11.63) | 0.418 |

Adjusted for sex, age category, nationality, place of death, season, and manner of death.

**Supplementary** **Table S9.** Odds ratio (OR) with 95% confidence interval for demographic and circumstantial characteristics.

|  |  |  | ***Crude*** |  |  | ***Adjusted*** |  |
| --- | --- | --- | --- | --- | --- | --- | --- |
| Antihypertensives  n=41/289 | ***Y/N*** | ***OR*** | ***95% CI*** | ***P value*** | ***OR*** | ***95% CI*** | ***P value*** |
| ***Sex*** |  |  |  |  |  |  |  |
| Male | 29/205 | 1.00 | - |  | 1.00 | - |  |
| Female | 12/84 | 1.01 | (0.49-2.07) | 0.979 | 0.98 | (0.43-2.23) | 0.952 |
| ***Age*** |  |  |  |  |  |  |  |
| ≤ 30 years | 0/26 | - | - |  | - | - |  |
| > 30 ≤ 60 years | 22/184 | 0.50 | (0.25-0.97) | 0.040 | 0.39 | (0.18-0.83) | 0.15 |
| > 60 years | 19/79 | 1.00 | - |  | 1.00 | - |  |
| ***Nationality*** |  |  |  |  |  |  |  |
| Italians | 25/215 | 1.00 | - |  | 1.00 | - |  |
| Non-Italians | 16/74 | 1.86 | (0.94-3.67) | 0.074 | 2.27 | (1.05-4.89) | 0.036 |
| ***Place of death*** |  |  |  |  |  |  |  |
| House | 19/190 | 1.00 | - |  | 1.00 | - |  |
| Public space | 15/58 | 2.59 | (1.24-5.41) | 0.012 | 3.07 | (1.36-6.97) | 0.007 |
| Health facility | 2/21 | 0.95 | (0.21-4.38) | 0.950 | 1.15 | (0.24-5.54) | 0.866 |
| Workplace | 4/9 | 4.44 | (1.25-15.81) | 0.021 | 5.06 | (1.27-20.16) | 0.022 |
| Place of confinement | 1/11 | 0.91 | (0.11-7.43) | 0.929 | 1.05 | (0.11-9.91) | 0.963 |
| ***Season*** |  |  |  |  |  |  |  |
| Spring | 13/70 | 1.00 | - |  | 1.00 | - |  |
| Summer | 10/85 | 0.63 | (0.26-1.53) | 0.311 | 0.65 | (0.25-1.67) | 0.366 |
| Autumn | 6/59 | 0.55 | (0.20-1.53) | 0.251 | 0.57 | (0.19-1.69) | 0.310 |
| Winter | 12/75 | 0.86 | (0.37-2.01) | 0.731 | 0.96 | (0.38-2.41) | 0.935 |
| ***Manner of death*** |  |  |  |  |  |  |  |
| Natural Cause | 22/148 | 1.00 | - |  | 1.00 | - |  |
| Accidental | 12/100 | 0.81 | (0.38-1.71) | 0.575 | 0.66 | (0.29-1.51) | 0.326 |
| Suicide | 5/30 | 1.12 | (0.39-3.20) | 0.830 | 1.00 | (0.32-3.18) | 0.995 |
| Homicide | 2/11 | 1.22 | (0.25-5.89) | 0.802 | 1.02 | (0.08-5.35) | 0.985 |

Adjusted for sex, age category, nationality, place of death, season, and manner of death.

**Supplementary** **Table S10.** Odds ratio (OR) with 95% confidence interval for demographic and circumstantial characteristics.

|  |  |  | ***Crude*** |  |  | ***Adjusted*** |  |
| --- | --- | --- | --- | --- | --- | --- | --- |
| Opioids  53/277 | ***Y/N*** | ***OR*** | ***95% CI*** | ***P value*** | ***OR*** | ***95% CI*** | ***P value*** |
| ***Sex*** |  |  |  |  |  |  |  |
| Male | 39/195 | 1.00 | - |  | 1.00 | - |  |
| Female | 14/82 | 0.85 | (0.44-1.66) | 0.640 | 0.98 | (0.47-2.06) | 0.960 |
| ***Age*** |  |  |  |  |  |  |  |
| ≤ 30 years | 2/24 | 1.00 | - |  | 1.00 | - |  |
| > 30 ≤ 60 years | 37/169 | 2.63 | (0.59-11.61) | 0.203 | 3.05 | (0.63-14.72) | 0.165 |
| > 60 years | 14/277 | 2.00 | (0.42-9.42) | 0.381 | 3.21 | (0.61-16.91) | 0.169 |
| ***Nationality*** |  |  |  |  |  |  |  |
| Italians | 36/204 | 1.00 | - |  | 1.00 | - |  |
| Non-Italians | 17/73 | 1.32 | (0.70-2.49) | 0.392 | 1.00 | (0.48-2.06) | 0.993 |
| ***Place of death*** |  |  |  |  |  |  |  |
| House | 32/177 | 1.00 | - |  | 1.00 | - |  |
| Public space | 8/65 | 0.68 | (0.30-1.55) | 0.361 | 0.58 | (0.24-1.41) | 0.230 |
| Health facility | 4/19 | 1.16 | (0.37-3.65) | 0.794 | 1.04 | (0.32-3.41) | 0.945 |
| Workplace | 2/11 | 1.01 | (0.21-4.75) | 0.994 | 0.73 | (0.14-3.68) | 0.699 |
| Place of confinement | 7/5 | 7.74 | (2.31-25.91) | 0.001 | 7.69 | (1.67-27.84) | 0.007 |
| ***Season*** |  |  |  |  |  |  |  |
| Spring | 18/65 | 1.00 | - |  | 1.00 | - |  |
| Summer | 16/79 | 0.73 | (0.35-1.55) | 0.413 | 0.92 | (0.41-2.06) | 0.845 |
| Autumn | 9/56 | 0.58 | (0.24-1.39) | 0.224 | 0.79 | (0.31-2.02) | 0.622 |
| Winter | 10/77 | 0.47 | (0.20-1.09) | 0.077 | 0.55 | (0.22-1.35) | 0.194 |
| ***Manner of death*** |  |  |  |  |  |  |  |
| Natural Cause | 24/146 | 1.00 | - |  | 1.00 | - |  |
| Accidental | 25/87 | 1.75 | (0.94-3.25) | 0.077 | 2.01 | (1.02-3.96) | 0.043 |
| Suicide | 2/33 | 0.37 | (0.08-1.64) | 0.190 | 0.39 | (0.09-1.80) | 0.228 |
| Homicide | 2/11 | 1.11 | (0.23-5.30) | 0.900 | 1.10 | (0.22-5.50) | 0.906 |

Adjusted for sex, age category, nationality, place of death, season, and manner of death.

**Supplementary** **Table S11.** Odds ratio (OR) with 95% confidence interval for demographic and circumstantial characteristics.

|  |  |  | ***Crude*** |  |  | ***Adjusted*** |  |
| --- | --- | --- | --- | --- | --- | --- | --- |
| CO  14/316 | ***Y/N*** | ***OR*** | ***95% CI*** | ***P value*** | ***OR*** | ***95% CI*** | ***P value*** |
| ***Sex*** |  |  |  |  |  |  |  |
| Male | 7/227 | 1.00 | - |  | 1.00 | - |  |
| Female | 7/89 | 2.55 | (0.87-7.48) | 0.088 | 1.82 | (0.59-5.62) | 0.301 |
| ***Age*** |  |  |  |  |  |  |  |
| ≤ 30 years | 0/26 | - | - |  | - | - |  |
| > 30 ≤ 60 years | 6/200 | 0.34 | (0.11-1.00) | 0.050 | 0.21 | (0.10-1.02) | 0.053 |
| > 60 years | 8/90 | 1.00 | - |  | 1.00 | - |  |
| ***Nationality*** |  |  |  |  |  |  |  |
| Italians | 8/232 | 1.00 | - |  | 1.00 | - |  |
| Non-Italians | 6/84 | 2.07 | (0.70-6.25) | 0.189 | 2.89 | (0.92-9.14) | 0.070 |
| ***Place of death*** |  |  |  |  |  |  |  |
| House | 14/195 | 1.00 | - |  | 1.00 | - |  |
| Public space | 0/73 | - |  |  |  |  |  |
| Health facility | 0/23 | - |  |  |  |  |  |
| Workplace | 0/13 | - |  |  |  |  |  |
| Place of confinement | 0/12 | - |  |  |  |  |  |
| ***Season*** |  |  |  |  |  |  |  |
| Spring | 5/78 | 1.00 | - |  | 1.00 | - |  |
| Summer | 0/95 | - |  |  |  |  |  |
| Autumn | 1/64 | 0.24 | (0.03-2.14) | 0.203 |  |  |  |
| Winter | 8/79 | 1.58 | (0.50-5.04) | 0.440 |  |  |  |
| ***Manner of death*** |  |  |  |  |  |  |  |
| Natural Cause | 0/170 | - | - |  | 1.00 | - |  |
| Accidental | 13/99 | 4.46 | (0.56-35.41) | 0.157 |  |  |  |
| Suicide | 1/34 | 1.00 | - |  |  |  |  |
| Homicide | 0/13 |  |  |  |  |  |  |

Adjusted for sex, age category, and nationality.

**Supplementary Figure S1**. Co-occurrence rates of the investigated classes of substances. Values are percentage of subjects with positive toxicological findings divided by both main classes and by increasing number of classes retrieved together, from one class only (N=1) up to 6 classes of substances at the same time.

**Supplementary Figure S2.** Distribution of number of prescribed drugs in positive cases divided by sex (males N=234, females N=96).

**Supplementary Figure S3.** Distribution of number of prescribed drugs in positive cases divided by age categories (<30 years N=26, ≥30-60 years N=206, ≥60 years N=98).
